# Detecting COVID-19 in Chest X-Ray Images via MCFF-Net

**DOI:** 10.1155/2021/3604900

**Published:** 2021-06-18

**Authors:** Wei Wang, Yutao Li, Ji Li, Peng Zhang, Xin Wang

**Affiliations:** ^1^School of Computer and Communication Engineering, Changsha University of Science and Technology, Changsha 410114, China; ^2^School of Electronics and Communications Engineering, Sun Yat-Sen University, Shenzhen 518107, China

## Abstract

COVID-19 is a respiratory disease caused by severe acute respiratory syndrome coronavirus (SARS-CoV-2). Due to the rapid spread of COVID-19 around the world, the number of COVID-19 cases continues to increase, and lots of countries are facing tremendous pressure on both public and medical resources. Although RT-PCR is the most widely used detection technology with COVID-19 detection, it still has some limitations, such as high cost, being time-consuming, and having low sensitivity. According to the characteristics of chest X-ray (CXR) images, we design the Parallel Channel Attention Feature Fusion Module (PCAF), as well as a new structure of convolutional neural network MCFF-Net proposed based on PCAF. In order to improve the recognition efficiency, the network adopts 3 classifiers: 1-FC, GAP-FC, and Conv1-GAP. The experimental results show that the overall accuracy of MCFF-Net66-Conv1-GAP model is 94.66% for 4-class classification. Simultaneously, the classification accuracy, precision, sensitivity, specificity, and F1-score of COVID-19 are 100%. MCFF-Net may not only assist clinicians in making appropriate decisions for COVID-19 diagnosis but also mitigate the lack of testing kits.

## 1. Introduction

Coronavirus disease 2019 (COVID-19) is a respiratory disease caused by severe acute respiratory syndrome coronavirus (SARS-CoV-2). Since its discovery in December 2019, the disease has spread rapidly around the world and is highly infectious. On March 11, 2020, the disease was declared a global pandemic by the World Health Organization (WHO) [1]. With the reopening of daily activities in countries around the world, the morbidity and mortality of COVID-19 have continued to increase, putting tremendous pressure on medical institutions and medical resources. Therefore, finding a quick and effective diagnosis method has become a top priority.

The current mainstream COVID-19 diagnosis technology is real-time reverse transcription polymerase chain reaction (RT-PCR) technology. However, the detection process is cumbersome and the diagnosis result has a high false-negative rate [2]. At the same time, chest imaging examinations, such as computed tomography (CT) and chest X-ray detection, also play a vital role in the early diagnosis of the disease [3]. Although the diagnostic efficiency of COVID-19 is constantly improving, the current cost of testing and diagnosis is still at a relatively high level. By examining the patient's lung imaging images, the diagnosis efficiency of COVID-19 can be greatly accelerated, and the patient can be treated as soon as possible.

Some studies have shown that COVID-19 has obvious clinical imaging characteristics. The study of Zu et al. [2] showed that some patients with COVID-19 had lung opacity in chest CT images. Zhao et al. [4] proposed that most patients have ground glass opacity (GGO), and some patients have lung consolidation and vasodilatation in chest lesions. Li and Xia [5] proposed that the CT imaging lesions of COVID-19 patients showed signs of GGO, lung consolidation, thickened interlobular septa, and air bronchography. Compared with CT, chest X-Ray (CXR) diagnosis has the advantages of convenient detection process, low cost, and low ionizing radiation intensity [6], which is more patient-friendly and easy to promote in remote and underdeveloped areas. In addition, manual image reading is a time-consuming and error-prone task. In order to reduce the pressure of medical imaging physicians, it is necessary to propose an efficient and accurate COVID-19 detection method.

In recent years, deep learning has become one of the most popular research fields in artificial intelligence. Deep convolutional neural network (DCNN) has excellent performance in computer vision tasks such as image classification, image segmentation, and target detection. A wealth of research results has emerged in this field. For example, Wang et al. [7] proposed and improved a deep learning method for detecting colon polyp images and achieved good results. Wang et al. [8] introduced the dense connection idea of the DenseNet model in the MobileNet model and proposed a new type of image classification model Dense-MobileNet. On the basis of the original model, the accuracy of the image classification task is improved and the complexity of the model is reduced. Wang et al. [9] combined the dense connection idea with the full convolutional network FCN model, proposed a dense full convolutional network DFCN, and used this model to perform semantic segmentation tasks on the Chenzhou remote sensing image dataset, achieving good results. After the outbreak of the COVID-19 epidemic, the use of DCNN to detect COVID-19 has become a current hot research field. At the same time, many outstanding research results have emerged in this field. Based on the characteristics of CXR images, Wang et al. [10] designed the Channel Feature Weight Extraction module (CFWE) and proposed a new network structure CFW-Net on this basis, which has achieved a good classification effect. Wang et al. [11] designed a Multiattention Interaction Enhancement module (MAIE) and proposed a new convolutional neural network, MAI-Net. The overall accuracy and COVID-19 category accuracy were 96.42% and 100%, respectively, which were better than those of ResNet [12]. Based on the VGG19 [13] network model, Apostolopoulos and Mpesiana [14] conducted a three-category classification experiment on a dataset containing COVID-19 positive, common pneumonia, and normal CXR images, and the overall classification accuracy rate was 93.48%. Wang et al. [15] proposed a COVID-Net network model based on the PEPX structure and introduced the depthwise separable convolution [16] into the network. The accuracy of the 3-class classification was 93.3%, which reduced the amount of model parameters and had good classification performance. Khan et al. [17] proposed a CoroNet network model based on the structure of Xception [18] and conducted 2-class, 3-class, and 4-class classification experiments for CXR images. The classification accuracy rates were 99%, 95%, and 89.6%. On this basis, Hussain et al. [19] improved Khan's work and proposed the CoroDet network structure. The classification accuracy of 2-class, 3-class, and 4-class were 99.1%, 94.2%, and 91.2%, respectively.

Unlike conventional image classification tasks, CXR images have high interclass similarity and low intraclass variability. This kind of data characteristics can easily lead to model deviation and overfitting problems, reduce the generalization performance of the network, and increase the difficulty of image classification tasks. To solve these problems, the Parallel Channel Attention Feature Fusion Module (PCAF) is designed. Based on the PCAF module, a new convolutional neural network structure, MCFF-Net, is proposed. MCFF-Net is used to perform a 4-class classification experiment on a dataset containing four types of image of COVID-19, normal, bacterial pneumonia, and viral pneumonia, with excellent performance. Compared with the deep learning methods in other documents, MCFF-Net has higher classification accuracy and stronger generalization ability.

## 2. CNNs

**Figure 1:**
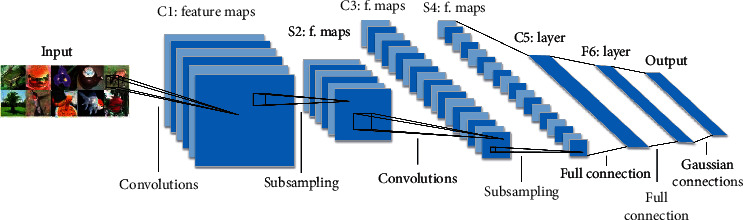
The basic structure of convolutional neural network [25].

In recent years, deep convolutional neural networks have been widely used in the field of computer vision, and its basic structure is shown in Figure 1. In view of the brand-new techniques such as ReLU [20], LRN [20], and Dropout [21], AlexNet [22] designed by Hintion and AlexKrizhevsky won the championship in 2012 ImageNet Challenge, with excellent performance. At the same time, AlexNet reduces the problem of network overfitting and enhances the generalization ability of the model. In 2014, Simonyan and Zisserman proposed the visual geometry group network (VGGNet) [14], which increased the network depth to 19 layers by alternately using 3 × 3 convolution kernels and 2 × 2 maximum pooling layers, significantly improving the network performance. Christian Szegedy et al. [23] designed the Inception module and constructed the GoogLeNet network based on this module. By increasing the width and depth, GoogLeNet also improves the utilization of the internal resources of the network and alleviates the problem of overfitting to a certain extent.

Increasing the network depth can improve network performance, but it can also cause some problems such as overfitting, network degradation, gradient disappearance, and gradient explosion. In 2015, He et al. [12] proposed the residual network named ResNet, which solved the degradation problem of the network through skip connection and increased the network depth to 1000 layers for the first time, making the deep convolutional neural network reach an unprecedented depth. Inspired by the residual network, the dense network named DenseNet was proposed by Huang et al. [24] in 2017 based on the idea of dense connections. By directly introducing short connections in any two layers to realize the reuse of features, it greatly reduces the amount of network parameters and effectively alleviates the problem of gradient disappearance of deep network.

## 3. PCAF Module

**Figure 2:**
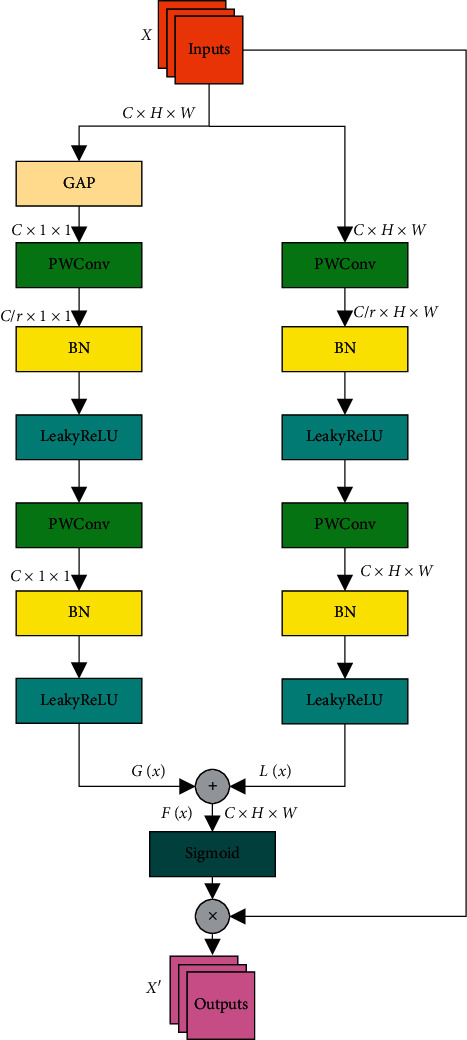
The structure of PCAF module.

In order to relieve the pressure of current medical staff and improve the diagnostic speed of COVID-19, we adopt a convolutional neural network that can adaptively learn the feature information exhaustively to identify and classify CXR images. CXR images have high interclass similarity and low intraclass variability. These problems will lead to model deviation and overfitting as well as reduce the recognition ability and generalization performance of the network. Hence, the PCAF module has been designed, whose structure is shown in Figure 2. C is the number of channels related to the input feature map. H and W represent the height and width of the feature map, respectively. “r” represents the channel compression ratio. “GAP” [26] represents the global average pooling. “PWConv” represents the 1 × 1 pointwise convolution. “BN” [27] is on behalf of batch normalization. “LeakyReLU” [28] and “Sigmoid” are activation functions. “⊕” represents the feature matrix bitwise addition operation. “⊗” represents the feature matrix bitwise multiplication operation.

The PCAF module is composed of two parallel branches, namely, the global feature extraction branch and the local feature extraction branch. The input feature map is imported into the two branches for feature extraction. Local feature extraction branch is composed of two PWConvs. The size of convolution kernel for the first PWConv is *C*/*r* × 1 × 1, compressing the channels of feature map to *C*/*r*, reducing the dimension of the feature map. The size of the second PWConv convolution kernel is *C* × 1 × 1, restoring the channels of the feature map to*C*, raising the dimension of the feature map.

Based on the above, the global feature extraction branch consists of one GAP layer and two PWConv layers. The GAP operation can compress the global information into a real number, which has the receptive field of global information to a certain extent.

Therefore, the global feature extraction branch focuses on extracting widely distributed global information in the feature map. The size of feature map in local feature extraction branch remains *H* × *W*. It has not been compressed by the global average pooling from beginning to end. Consequently, more attention is paid to extract the local subtle information of the feature map.

The output features of the two branches can be expressed as

(1) $$\begin{array}{c} G\;\left(X\right)=d\left\{\text{BN}\left[\text{PWConv}2\left[d\left[\text{BN}\left[\text{PWConv}1\left[\text{GAP}\left(X\right)\right]\right]\right]\right]\right]\right\},\\ L\;\left(X\right)=d\left\{\text{BN}\left[\text{PWConv}2\left[d\left[\text{BN}\left[\text{PWConv}1\left(X\right)\right]\right]\right]\right]\right\}, \end{array}$$

where ΒN represents batch normalization operation and *d* represents LeakyReLU activation function.

After output features *L*(*X*) and *G*(*X*) of the two branches are fused by matrix bitwise addition operation, the fusion feature *F*(*X*) is obtained by the sigmoid activation function, which can be described by the following formula:

(2) $$\begin{array}{c} F\left(X\right)=G\left(X\right)\;⊕\;L\left(X\right). \end{array}$$

The features of different scales are merged by *F*(*X*). In this way, the weight of each channel in the feature map is recalibrated. The model can learn the weight coefficient of each channel in the global feature and the weight coefficient of each channel in the local feature, respectively.

Finally, the mask *F*(*X*) and the input feature map *X* are processed by matrix bitwise multiplication operation, and the output feature map *X*′ is obtained. The formula is as follows:

(3) $$\begin{array}{c} X^{\prime }=X⊗\sigma \left(F\left(X\right)\right)=X⊗\sigma \left[\left(L\left(X\right)\;⊕\;G\left(X\right)\right)\right], \end{array}$$

where *σ* represents Sigmoid activation function.

After the input feature map is processed by the PCAF module, the network can learn more important information in a targeted manner, ignoring the secondary information.

## 4. MCFF-NET

**Figure 3:**
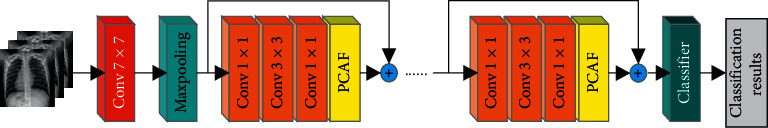
The network structure of MCFF-Net.

Based on the PCAF module, three convolutional neural networks with different depths are proposed: Multiscale Channel Feature Fusion Network (MCFF-Net), as shown in Table 1. When calculating the network depth in Table 1, a PCAF module is recorded as one layer, and the depth of the classifier in MCFF-Net is uniformly recorded as one layer. The “Conv” structure in Table 1 can be expressed as a composite structure including “convolution,” “batch normalization”, and “ReLU activation function.” The value after “Conv” represents the number of channels corresponding to the structure. The network diagram is shown in Figure 3.

In traditional convolutional neural networks such as AlexNet [22] and VGGNet [14], three fully connected layers (3 full connection layer, 3-FC) are used as classifiers. This can increase the nonlinear expression ability of network, accompanied by a large amount of memory occupation and high calculation overhead, which has caused a substantial increase in the amount of network parameters. In order to reduce the network parameters, our network uses a fully connected layer (1-FC) as the classifier to convert the computational overhead of the image recognition task to the convolutional layer, which reduces the burden of the fully connected layer.

Due to the extremely large number of features output by the convolutional layer, one fully connected layer as a classifier will cause excessive parameters. Therefore, we first reduce the output feature map size of the convolutional layer to 1 × 1 through the GAP operation and then classify through the fully connected layer, which greatly reduces the amount of parameter of the network model. “GAP-FC” is used to represent this structure.

Besides, 1 × 1 point convolution is considered to be inserted in front of the GAP structure, reducing the dimensionality of the output feature map at the end of the network. The classifier designed under this idea has nothing to do with the fully connected layer, thereby further reducing the amount of parameter. “Conv1-GAP” is used to represent this structure.

**Figure 4:**
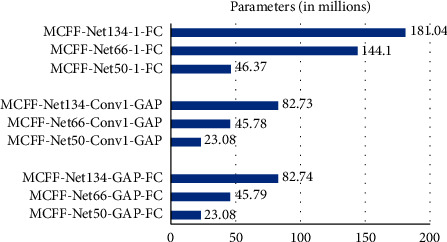
The parameter comparison of MCFF-Nets.

**Figure 5:**
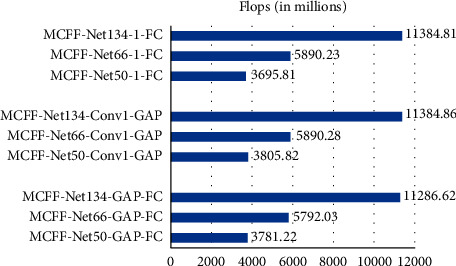
The comparison of floating points of operations (FLOPs).

When using different depth networks and different classifiers to recognize CXR images, there are differences among the amount of parameter and calculation of the network. Take the 4-class classification task as an example, and suppose the output feature map size of the last convolutional layer in the network is *H* × *W* × *D*. When using a fully connected layer “1 − FC” as the classifier, the parameter of the network is 4 × *H* × *W* × *D*+4. When the “GAP-FC” structure is used as the classifier, the parameter of the network is *D*+*D* × 4+4. When the “Conv1-GAP” structure is used as the classifier, the parameter of the network is *H* × *W* × 4+*D* × 4+4. When MCFF-Nets with different depths use different classifiers, the parameters are shown in Figure 4. Comparison of floating point of operations (FLOPs) is shown in Figure 5.

From Figure 4, the sort of classifier has great influence on the network parameters. In the case of the same network depth, the networks using the “1 − FC” classifier are obviously larger than those using other classifiers. Therefore, using the “1 − FC” classifier should be avoided as much as possible under the premise of ensuring the classification accuracy. In addition, the network depth also has a huge impact on the amount of network parameters. The parameters of MCFF-Net134-GAP-FC are 3.90 times that of MCFF-Net50-GAP-FC, and the parameters of MCFF-Net134-GAP-FC are 1.26 times that of MCFF-Net66-GAP-FC.

According to Figure 5, the computational cost is mainly determined by the depth of network. MCFF-Net134 is very computationally intensive. Compared with MCFF-Net66, MCFF-Net134 has a FLOPs increase of 94.87%. MCFF-Net66 has an increase of 53.18% compared to MCFF-Net50. Compared with MCFF-Net66, MCFF-Net134 has an increase of 194.87%, which is the largest increase in calculations. In conclusion, when there is no notable difference in recognition accuracy, in order to save computational cost, MCFF-Net66 has the highest cost performance.

## 5. Experiments and Results

### 5.1. Datasets

Since COVID-19 is a new type of disease, there is a lack of datasets suitable for this study. In this paper, we have constructed a dataset by collecting CXR images from public image databases.

In order to further evaluate the generalization performance of the MCFF-Net, a 4-class dataset has been constructed. Dataset collects CXR images from five different public databases. These databases are (1) Actualmed-COVID-chestxray-dataset [29]; (2) COVID-19 Radiography Database [30]; (3) Figure 1-COVID-chestxray-dataset [31]; (4) Pneumonia Virus vs. Pneumonia Bacteria [32]; and (5) Chest X-ray Image [33]. Dataset contains four classes of CXR images, namely, COVID-19, normal, bacterial pneumonia, and viral pneumonia, totaling 5,985 images. There are 5300 images in training sets, including 800 COVID-19 patient images, 1300 normal images, 1600 viral pneumonia images, and 1600 bacterial pneumonia images. There are 741 images in the test sets, including 142 images of COVID-19 patients, 200 normal images, 202 bacterial pneumonia images, and 197 viral pneumonia images.

**Figure 6:**
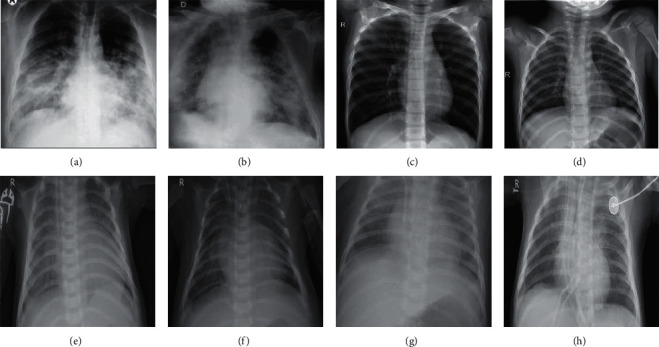
Chest X-ray images. (a) COVID-19, (b) COVID-19, (c) normal, (d) normal, (e) pneumonia—bacteria, (f) pneumonia—bacteria, (g) pneumonia—viral and (h) pneumonia—viral.

The eight sample images from the dataset that we have established are shown in Figure 6.

### 5.2. Experimental Setup

The experiments are carried out on the same platform and environment to ensure the credibility of the comparison results between different network models.  Table 2 shows the software and hardware configuration information of the experimental platform. The batch size of the training set and the test set is both 16.

The learning rate annealing algorithm is introduced in the training process, and a larger learning rate is used in the initial stage of training. As the number of iterations increases, the learning rate is gradually reduced. This algorithm can avoid large fluctuations of classification accuracy in the later stage of training, so as to get closer to the optimal solution. After repeated experiments, we finally adjusted the parameter settings as follows: the initial learning rate is set to 0.001. Since the first 50 epochs, the learning rate decays twice as much as before and then decreases by 2 times every 50 epochs. A total of 300 epochs are used for training. In order to evaluate the performance of the model more objectively, we take the recognition accuracy of the last 10 epochs on test set to calculate the average value, which is used as the final classification accuracy.

### 5.3. Evaluation Criteria

In this section, we will explain the evaluation indicators used to quantify the classification performance of the network: accuracy, precision, sensitivity, specificity, and *F*1-score. In order to represent the above indicators, we also need to count the four numbers in the confusion matrix: true positive (TP), true negative (TN), false positive (FP), and false negative (FN).

### 5.4. Experimental Results and Discussion

**Figure 7:**
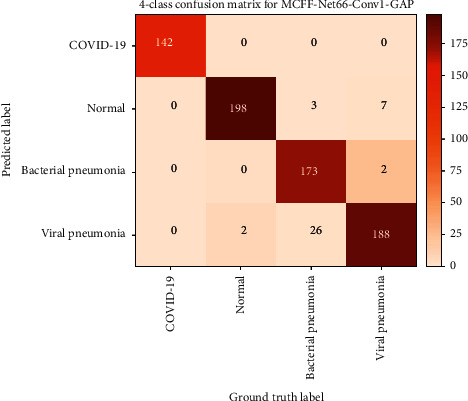
The 4-class confusion matrix of the MCFF-Net66-Conv1-GAP.

**Figure 8:**
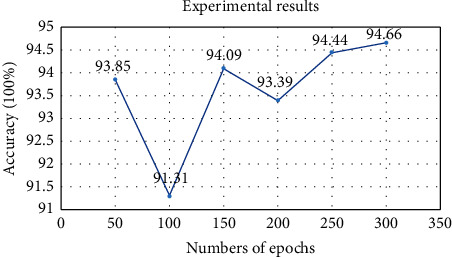
The 4-class accuracy rate of MCFF-Net66-Conv1-GAP.

In order to further verify the generalization ability of MCFF-Net66-Conv1-GAP in the CXR image recognition task, we increase the difficulty of the classification task and use the model to conduct experiments on dataset with four classes of CXR images. The training period is 300 epochs, which is divided into 6 stages, each with 50 epochs. We take test set recognition accuracy of the last 5 epochs in each stage and calculate the average value as the experimental result of the corresponding stage.  Figure 7 shows the 4-class confusion matrix of MCFF-Net66-Conv1-GAP.  Figure 8 shows the overall accuracy of MCFF-Net66-Conv1-GAP in each stage of the 4-classification experiment.

According to Figures 7 and 8, the overall accuracy of the four classification tasks of MCFF-Net66-Conv1-GAP reaches 94.6%, showing that the MCFF-Net has excellent classification performance in CXR image recognition tasks. In the discussion of Introduction, we briefly described a variety of COVID-19 detection methods proposed by researchers from various regions of the world. Some models are suitable for 2-class classification, and some models are suitable for multiclass classification. Hence, the model MCFF-Net66-Conv1-GAP is compared with the methods of Khan [17], Hussain [19], Mangal [34], and Joshi [35]. The comparison results are shown in Table 3.

According to Table 4, our proposed network model MCFF-Net66-Conv1-GAP can efficiently help classify CXR images of COVID-19-positive patients, normal, and ordinary pneumonia patients. What is more, the overall accuracy, sensitivity, specificity, and *F*1-score of COVID-19 images have reached 100%.

The various methods in Table 3 use different numbers of CXR images from different data sources for training. The number of images used for training is shown in the fourth column. When there are four values in the number of images column, then the first value indicates the number of COVID-19 images, the second value indicates the number of viral pneumonia images, the third value indicates the number of bacterial pneumonia images, and the fourth value indicates the number of normal images. “N/A” indicates an item of information that is not disclosed in the abovementioned documents.

Compared with other methods, we have used the largest number of COVID-19 images to train our MCFF-Net model and have got 94.66% classification accuracy in the 4-class recognition task, which are higher than other methods in Table 3. This shows that the MCFF-Net has better performance in CXR image classification tasks.

### 5.5. Experimental Analysis

According to the experimental results in Section 5.4, we can find that, in the 4-class classification experiments, MCFF-Net66-Conv1-GAP has been chosen to conduct a 4-class classification experiment. The experimental results are compared with other existing methods. The overall accuracy of the 4-class classification experiment is 94.66%. In conclusion, the overall performance is better than other existing methods.

Through experimental analysis, it can be seen that, in the CXR image classification task of COVID-19, the network depth should be kept moderate. If the network is too shallow, it is hard to fully extract the feature information. If the network is too deep, while greatly increasing the amounts of parameters and calculations, it is also likely to overfitting and gradient explosion problems.

Because CXR images have high similarity between classes and low intraclass variability, it is easy to cause model deviation and overfitting, which increases the difficulty of image classification tasks. Therefore, this paper designs a PCAF module, which is composed of two parallel branches, and includes “GAP” and “PWConv” structures. After the input feature map is processed by the PCAF module, the output feature map will both have global and local information in the image, which improves the feature extraction capability of the network.

## 6. Conclusions

In this paper, a Parallel Channel Attention Feature Fusion (PCAF) module is designed according to the characteristics of CXR images. And based on this module, a new convolutional neural network structure MCFF-Net is proposed to classify CXR images in order to diagnose and detect COVID-19 cases. Through the analysis and comparison of the experimental results, we believe that MCFF-Net66-Conv1-GAP has the highest application value. The overall accuracy of the 4-class classification experiment and the COVID-19 image recognition accuracy have reached 94.66% and 100%, respectively. Despite the fact that good results have been achieved, MCFF-Net still needs clinical research and testing. We will overcome the limitations of hardware conditions and train the MCFF-Net with a larger dataset to further improve its classification accuracy.

## Figures and Tables

**Table 1:** MCFF-Net configuration.

MCFF-Net50		MCFF-Net66		MCFF-Net134	
Conv7 × 7-64, stride 2					
3 × 3 maxpooling, stride 2					
Conv3 × 3-64	×3	Conv1 × 1-64	×3	Conv1 × 1-64	×3
Conv3 × 3-64		Conv3 × 3-64		Conv3 × 3-64	
*PCAF-64*		Conv1 × 1-256		Conv1 × 1-256	
		*PCAF-256*		*PCAF-256*	
Conv3 × 3-128	×4	Conv1 × 1-128	×4	Conv1 × 1-128	×4
Conv3 × 3-128		Conv3 × 3-128		Conv3 × 3-128	
*PCAF-128*		Conv1 × 1-512		Conv1 × 1-512	
		*PCAF-512*		*PCAF-512*	
Conv3 × 3-256	×6	Conv1 × 1-256	×6	Conv1 × 1-256	×23
Conv3 × 3-256		Conv3 × 3-256		Conv3 × 3-256	
*PCAF-256*		Conv1×1-1024		Conv1×1-1024	
		*PCAF-1024*		*PCAF-1024*	
Conv3 × 3-512	×3	Conv1 × 1-512	×3	Conv1 × 1-512	×3
Conv3 × 3-512		Conv3 × 3-512		Conv3 × 3-512	
*PCAF-512*		Conv1 × 1-2048		Conv1 × 1-2048	
		*PCAF-2048*		*PCAF-2048*	
Average pooling					
Classifier, softmax					

**Table 2:** Experimental platform configuration.

Attributes	Configuration information
Operating system	Ubuntu 18.04.5 LTS
CPU	Intel (R) Xeon (R) silver 4214 CPU @ 2.20 GHz
GPU	GeForce RTX 2080
CUDNN	CUDNN 7.5.0
CUDA	CUDA 10.0.130
Frame	Fastai
IDE	PyCharm
Language	Python

**Table 3:** Accuracy comparison of our proposed method with other existing deep learning methods.

Study	Architecture	Accuracy 4-class (%)	Number of images	# of parameters (in millions)
Khan et al. [17]	CoroNet	89.60	284, 327, 330, 310	33
Hussain et al. [19]	CoroDet	91.20	500, 400, 400, 800	N/A
Mangal et al. [34]	CovidAID	87.20	115, 1337, 2530, 1341	N/A
Joshi et al. [35]	DarkNet-53	76.46	659, 1493, 2772, 1660	N/A
Proposed method	MCFF-Net	94.66	800, 1600, 1600, 1300	45.78

**Table 4:** Average class-wise accuracy, precision, recall, specificity, *F*1-score of 4-class MCFF-Net66-Conv1-GAP (%).

Class	Accuracy (%)	Precision (%)	Recall (%)	Specificity (%)	*F*1-score (%)
COVID-19	100	100	100	100	100
Normal	98.38	99	95.19	99.63	97.06
Viral pneumonia	95.82	85.64	98.86	94.88	91.78
Bacterial pneumonia	95.01	95.43	87.04	98.29	91.04
Average	97.3	95.02	95.27	98.2	94.79

## Data Availability

All datasets in this article are public datasets and can be found respectively in references [29–33].
